# Accumulation of humic-like fluorescent dissolved organic matter in the Japan Sea

**DOI:** 10.1038/srep05292

**Published:** 2014-07-16

**Authors:** Kazuki Tanaka, Kenshi Kuma, Koji Hamasaki, Youhei Yamashita

**Affiliations:** 1 Graduate School of Environmental Science, Hokkaido University; 2 Faculty of Fisheries Science, Hokkaido University; 3 Atomosphere and Ocean Research Institute, The University of Tokyo; 4 Faculty of Environmental Earth Science, Hokkaido University

## Abstract

Major fraction of marine dissolved organic matter (DOM) is biologically recalcitrant, however, the accumulation mechanism of recalcitrant DOM has not been fully understood. Here, we examine the distributions of humic-like fluorescent DOM, factions of recalcitrant DOM, and the level of apparent oxygen utilization in the Japan Sea. We find linear relationships between these parameters for the deep water (>200 m) of the Japan Sea, suggesting that fluorescent DOM is produced in situ in the Japan Sea. Furthermore, we find that the amount of fluorescent DOM at a given apparent oxygen utilization is greater in the deep water of the Japan Sea than it is in the North Pacific, where the highest level of fluorescent DOM in the open ocean was previously observed. We conclude that the repeated renewal of the deep water contributes to the accumulation of fluorescent DOM in the interior of the Japan Sea.

At 662 Pg C, oceanic dissolved organic matter (DOM) is a huge pool of reduced carbon, holding greater than 200 times the carbon inventory of marine biomass^1^. The majority of oceanic DOM is considered to be of marine origin, and the microbial production of DOM (defined as the microbial carbon pump) has been implicated as one of the processes that produce recalcitrant DOM^2^,^3^. Thus, it can be hypothesized that microbially derived, recalcitrant DOM accumulates in oceans. It has been hypothesized that oceanic DOM pool has been historically dynamic and that the changes in its size and the associated sequestration of CO_2_ (at least 1600 PgC) or release of CO_2_ from its degradation have affected the state of paleoclimates^4^. However, because the annual to decadal variation in the amount of recalcitrant DOM in oceans may be too small to detect, we do not know whether the present oceanic DOM pool is stable or dynamic^5^.

Though dissolved organic carbon (DOC) analyses can detect relatively small differences in DOM quantity due to recent improvements in analytical capabilites^1^,^6^, DOC concentrations alone do not provide insights into the composition of DOM, which is useful for evaluating DOM dynamics^6^,^7^,^8^,^9^. One of the more sensitive analytical methods for detecting DOM composition is the fluorescence technique^10^,^11^,^12^. Though this technique can only evaluate fluorescent fractions in DOM, two major types of fluorophores, i.e., protein-like fluorophores (FDOM_A_) and humic-like fluorophores (FDOM_H_), can be detected using excitation-emission matrix (EEM) fluorescence^10^,^11^,^12^,^13^,^14^. It is well documented that a major source of FDOM_H_ in coastal environments is the riverine inputs of terrestrial FDOM_H_^11^. Conversely, FDOM_H_ can also be produced during the microbial degradation of organic matter^15^,^16^,^17^. Although FDOM_H_ is known to be easily degraded by sunlight^18^,^19^,^20^, linear relationships between the fluorescence intensity of FDOM_H_ and indicators of microbial remineralization, namely, the apparent oxygen utilization (AOU), have been observed throughout deep oceans globally^7^,^21^,^22^,^23^,^24^,^25^. In addition, the linear relationships between the amounts of FDOM_H_ and AOU present in the bathypelagic layer throughout the Pacific indicate that in situ-produced FDOM_H_ is bio-recalcitrant over the time scale of global thermohaline circulation (at least 900 years)^7^. These experimental and observational results suggest that FDOM_H_ is a product of the microbial carbon pump and is thus a useful indicator for evaluating DOM dynamics, especially its recalcitrant fraction.

The Japan Sea is a marginal sea in the western North Pacific that is connected to the East China Sea, the western North Pacific, and the Okhotsk Sea, and it has sills that are shallower than 150 m (Fig. 1). The Japan Sea spans from subarctic to subtropical conditions and is affected by global oceanic processes, e.g., subduction and deep-water formation. There is a relatively uniform water mass at the depths below thermocline that is named the Japan Sea Proper Water (JSPW)^26^,^27^,^28^. The JSPW is known to contain high levels of oxygen and ^14^C of total dissolved inorganic carbon compared with other locations in the North Pacific and even the South Pacific, which is an indication of active deep convection^28^,^29^,^30^,^31^,^32^. Due to its semi-closed characteristics, the turnover time of the JSPW by convection and the residence time of the water within the Japan Sea have been considered separately and are estimated to be 100 and 1000 years, respectively^29^. The dissolved oxygen concentrations in the bottom layer of the JSPW have decreased during recent decades, indicating a weakening of the deep convection systems^31^.

Though only a few studies have clarified the distributional patterns of DOM in the Japan Sea, it was reported that DOC concentrations in the JSPW were higher than those in deep waters of the North Pacific^33^. Such high levels of DOC are possibly due to transport of semi-labile DOC from surface layers^1^ along with the JSPW formation. On the other hand, similar levels of FDOM_H_ between the JSPW and the deep waters of the North Pacific were also reported^34^. DOM in the deep waters of the North Pacific can be characterized as lowest DOC concentration^1^,^5^ and highest levels of recalcitrant FDOM_H_^7^,^24^ in the global deep ocean, indicative of the oldest DOM in the global ocean. Thus, there should be some specific mechanisms for keeping high levels of FDOM_H_ in the JSPW that age are significantly younger than the deep waters of the North Pacific^32^.

To obtain new insights into DOM dynamics in the JSPW as well as in the global ocean, we determined the spatial distributions of FDOM_H_ in the JSPW and in the deep waters of the western North Pacific (Fig. 1), two areas in which the levels of AOU differ significantly (Fig. 2). Water samples were collected from five sites in the Japan Sea (two in the Japan Basin and the others in the Yamato Basin) and five sites in the western North Pacific (Fig. 1). To evaluate the FDOM_H_ composition, filtered seawater samples were analyzed using EEM fluorescence and parallel factor analysis (PARAFAC)^14^,^35^.

## Results

### FDOM_H_ in the Japan Sea

A three-component model was validated based on the PARAFAC modeling of EEMs from the Japan Sea and the western North Pacific (Fig. 3). Components 1 (FDOM_H_-1) and 2 (FDOM_H_-2) exhibited fluorescence peaks in the region of humic-like fluorophores^10^,^11^,^12^,^13^,^14^. Component 3 (C3) had a peak with an excitation wavelength less than 300 nm and an emission wavelength less than 350 nm and was characterized as representing protein-like fluorophores^10^,^11^,^12^,^13^,^14^. Because we found a contamination peak that was similar to that of the protein-like fluorophores, C3 was considered to include the edge of the contamination peak and was thus not included in further analyses.

FDOM_H_-1 had a peak at the 470-nm emission wavelength and could be categorized as a humic-like fluorophore that is traditionally defined as terrestrial^10^,^11^. In contrast, FDOM_H_-2 could be assigned as a humic-like fluorophore that is traditionally defined as marine because this FDOM_H_ had a peak at the 395-nm emission wavelength^10^,^11^. Similar PARAFAC components to FDOM_H_-1 and FDOM_H_-2 have been reported in previous EEM-PARAFAC studies that examined the open ocean^23^,^24^,^36^,^37^ (Fig. 3).

**Figure 2:**
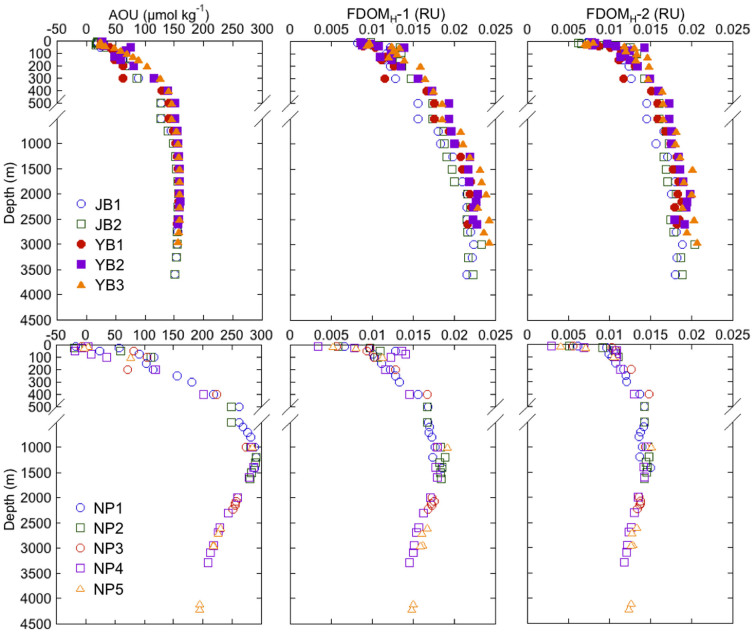
Vertical profiles of AOU, FDOM_H_-1, and FDOM_H_-2 in the Japan Sea (upper panels) and the western North Pacific (lower panels).

The vertical profiles of FDOM_H_-1 and FDOM_H_-2 were similar to each other in the Japan Sea, and this similarity was observed regardless of the sampling site examined (Fig. 2). The levels of FDOM_H_ were the lowest in surface waters, gradually increased with depth, and were relatively constant at depths greater than 2000 m (Fig. 2). Though EEM-PARAFAC was firstly applied for the JSPW in the present study, similar vertical distributions of FDOM_H_ were previously observed in the Japan Sea by measuring the fluorescence intensities at a single pair of excitation (320 nm) and emission (420 nm) wavelengths^34^,^38^ and by using EEM^33^. In the western North Pacific, the amounts of FDOM_H_-1 and FDOM_H_-2 were also lowest in the surface waters, followed by an increase with depth that peaked at 1000–1500 m and then decreased slightly with depth (Fig. 2). The vertical distribution of FDOM_H_ in the western North Pacific was similar to those previously observed in the North Pacific^7^,^23^. Though the highest levels of FDOM_H_ previously observed in the open ocean globally were recorded for the intermediate water of the North Pacific^7^,^24^, the amount of FDOM_H_-1 and FDOM_H_-2 measured in the JSPW was similar or slightly higher than that recorded in the intermediate water of the western North Pacific (Fig. 2). The vertical profiles of both FDOM_H_ were similar with those of AOU for the Japan Sea and the western North Pacific (Fig. 2).

### Relationships between FDOM_H_ and AOU

The JSPW was divided into upper and deeper waters at 1000 m based on the vertical structure of potential temperature^26^,^27^. Furthermore, the deeper JSPW can also be divided into two water masses at a depth of about 2000–2500 m^26^,^28^,^32^. According to these criteria, the deep water masses in the Japan Sea were divided and defined as follows: the upper JSPW (UJSPW, 200–1000 m), the Japan Sea Deep Water (JSDW, 1000–2000 m), and the Japan Sea Bottom Water (JSBW, 2000 m-bottom). The deep water masses of the western North Pacific were separated into intermediate water (200–1000 m) and North Pacific Deep Water (NPDW, 1000 m-bottom) according to Refs. (7, 23). Except in the JSBW which contained a very narrow range of AOU and FDOM_H_ values (Fig. 2), the FDOM_H_-1 and FDOM_H_-2 values were linearly correlated with the amount of AOU for the individual water masses of the Japan Sea and the western North Pacific at depths below 200 m, where the photobleaching of FDOM_H_ can be negligible (Fig. 4; Table 1). The relationships between FDOM_H_ and AOU differed between the Japan Sea and the western North Pacific. Notably, the amount of FDOM_H_ at a given level of AOU was considerably greater for the Japan Sea compared with the western North Pacific.

## Discussion

Similar to other oceans^7^,^21^,^22^,^23^,^24^,^25^, we find linear relationships between AOU and FDOM_H_ for the deep water (>200 m) of the Japan Sea (Fig. 4; Table 1), indicating that both the FDOM_H_-1 (traditionally defined as terrestrial humic-like DOM) and the FDOM_H_-2 (traditionally defined as marine humic-like DOM) are produced in situ in the JSPW as organic matter is oxidized biologically. This result is similar to previous findings obtained for the open ocean^23^,^24^. Interestingly, the relationships between FDOM_H_ and AOU differed between the Japan Sea and the western North Pacific, namely, the levels of FDOM_H_-1 and FDOM_H_-2 at a given AOU are greater for the Japan Sea compared with the western North Pacific. A possible cause for this difference in the FDOM_H_-AOU relationships is a difference in the production mechanism of FDOM_H_ in the ocean interior, i.e., a difference in the ratio of FDOM_H_ production to oxygen consumption. However, a single linear regression between FDOM_H_ and AOU could be fit to the data for the bathypelagic layer throughout the Pacific^7^. In addition, one general linear regression fit the relationship between the amount of the fluorescent DOM components (corresponding to FDOM_H_-1 and FDOM_H_-2) and the level of AOU for the global deep ocean^24^. These previous findings imply that the characteristics of the production mechanism of FDOM_H_ are very similar, regardless of differences in oceanic regions. On the other hand, recent long-term (>1year) experiments revealed that production of FDOM_H_ depends on the lability of the substrate; the larger the production of FDOM_H_ relative to oxygen consumption was found for the less labile substrate^15^. The chemical composition of sinking particles suggests that sinking particles at the deep layer is less labile compared with those at the intermediate layer^39^, thus, differences in FDOM_H_ production against lability of sinking particles might be reason of differences in FDOM_H_-AOU relationships among the UJSPW, JSDW, and JSBW (Fig. 4). Therefore, the difference between the FDOM_H_-AOU relationships of the Japan Sea and the western North Pacific is possibly attributed to other factors and not to a difference in the production mechanism of FDOM_H_, even though the difference found among the JSPW might be due to different lability of sinking particles.

Although a general FODM_H_-AOU linear relationship has been observed for the global ocean, the levels of FDOM_H_ found in the North Atlantic Deep Water (NADW) were relatively high compared with those expected based on the FDOM_H_-AOU relationship for the global ocean^24^. These deviations for the NADW have been attributed to the contribution of terrestrial FDOM_H_ derived from the Arctic Ocean because relatively high concentrations of lignin phenols, a unique terrestrial biomarker, were observed in the NADW^40^. In the case of the Japan Sea, the Tsushima Warmer Current, which carries freshwater from the East China Sea, affects the surface water chemistry^41^. Terrestrial DOM derived from the Yangtze River, in particular, has been known to distribute into the East China Sea^42^,^43^. In addition, freshwater from the Amur River has been known to flow into the northern part of the Japan Sea^44^. Thus, in addition to in situ-produced FDOM_H_, terrestrial DOM derived from the East China Sea and/or the Amur River might contribute to the surface water FDOM_H_ of the Japan Sea and thus to the JSPW.

In coastal environments, the shift of the peak position of FDOM_H_ to a shorter wavelength (blueshift) with an increase in salinity has been well documented^10^,^11^,^45^. Such changes in emission maxima have been considered a result of the shift in the major fluorescent component from a terrestrial (corresponding to FDOM_H_-1) to a marine origin (corresponding to FDOM_H_-2)^45^,^46^. In other words, though both FDOM_H_-1 and FDOM_H_-2 are produced at oceanic as well as terrestrial environments, oceanic and terrestrial FDOM is rich in FDOM_H_-2 and FDOM_H_-1, respectively. In case of the JSPW, a higher contribution of terrestrial FDOM_H_ would change the composition of the FDOM_H_ by increasing the ratio of FDOM_H_-1 to FDOM_H_-2. However, the ratio of FDOM_H_-1 to FDOM_H_-2 in the JSPW was similar to or even lower than that in the intermediate and deep waters of the western North Pacific (Fig. 5), suggesting that the contribution of terrestrial FDOM_H_ to the bulk FDOM_H_ signature in the JSPW is similar to or smaller than that in the western North Pacific. The ratio of FDOM_H_-1 to FDOM_H_-2 tended to increase with depth (Fig. 5). A blueshift associated with photobleaching has been observed for both coastal and oceanic FDOM_H_^18^,^19^, suggesting that the FDOM_H_ in shallower water masses is more photobleached compared with that found in deeper water masses and that the contribution of photobleached FDOM_H_ in the JSPW is potentially greater than that in the western North Pacific. Although the vertical patterns of FDOM_H_ composition cannot refute the possibility of the contribution of photobleached terrestrial FDOM_H_ to the JSPW, the FDOM_H_-AOU linear relationship (Fig. 4) and conservative behavior of terrestrial chromophoric DOM including FDOM_H_ in the Yangtze River estuary^42^,^47^ as well as the East China Sea^43^ indicate that the contribution of photobleached terrestrial FDOM_H_ is minor in the JSPW. The ratios measured in surface waters of the Japan Sea and the western North Pacific had large variations, suggesting that the contributions of terrestrial FDOM_H_ and/or autochthonously produced FDOM_H_ (and the corresponding organisms, including the marine phytoplankton^16^) in surface waters are different from those of the deep ocean.

Based on our findings and discussion above, the difference in the FDOM_H_-AOU relationships between the Japan Sea and the western North Pacific is potentially due to the process rather than the in situ production mechanism or the contribution of terrestrial FDOM_H_. The JSPW is formed in the Japan Sea and has an independent convection system. During the deep convection process (the formation of the JSPW), original JSPW, which is characterized by high levels of AOU and FDOM_H_ compared with surface waters, is transported to the surface layer; then, the level of the AOU decreases because of the mixing of JSPW with surface water and the supply of oxygen from the atmosphere. At the same time, the level of FDOM_H_ also decreases with mixing but is not extensively degraded by photobleaching because of the low winter insolation and the short residence time in the surface layer. It should be emphasized that the turnover time of the JSPW by convection is approximately 100 years, but its residence time within the Japan Sea is approximately 1000 years^29^, indicating that most of the water that is upwelled by deep convection returns to the deep layer within a relatively short time scale and does not flow out of the Japan Sea into the North Pacific or the Okhotsk Sea. Hence, the water mass, which is characterized by a relatively low AOU and a relatively high FDOM_H_, is subsequently redistributed as “new” JSPW. Thus, the in situ production of FDOM_H_ in the JSPW and the repeated renewal of the JSPW result in the accumulation of FDOM_H_ in the deep layer of the Japan Sea.

Although the amount of carbon in FDOM_H_ has not been clearly determined, FDOM_H_ may contribute to the high levels of DOC in the JSPW^33^. The present study elucidates a potential mechanism for the accumulation of FDOM_H_ in the deep ocean. Because FDOM_H_ is bio-recalcitrant on a time scale similar to that of thermohaline circulation^7^ and the FDOM_H_ pool in the open ocean is basically balanced by the rates of in situ production and photobleaching (and possibly contributions of terrestrial FDOM_H_), the results of the present study indicate that FDOM_H_ can accumulate in the ocean interior if this balance is upset by changes in processes such as the thermohaline circulation.

## Methods

Samples from the Japan Sea were collected at 2 stations (JB1, JB2) in the Japan Basin and 3 stations (YB1, YB2, YB3) in the Yamato Basin from November 5 to 9, 2007, as part of the T/S *Oshoro-maru* cruise (C184). Water samples from the western North Pacific were collected at 5 stations from July 30 to August 3, 2011 as part of the R/V *Tansei Maru* cruise (KT-11-17). Salinity and temperature were measured using a CTD, and dissolved oxygen concentrations were measured using an oxygen sensor (SBE 43) connected to a CTD. Seawater samples for fluorescence analyses were collected with Niskin bottles. An in-line filter that was attached directly to the spigot of a Niskin bottle was used for filtration, and the seawater was gravity filtered. Acid-washed 0.22-μm filters (Millipak 100 cartridge, Millipore) and pre-combusted glass fiber filters (GF/F, Whatman) were used to filter the Japan Sea and the western North Pacific samples, respectively. Thus, FDOM_H_ levels in the Japan Sea may be slightly underestimated compared with those in the western North Pacific. Filtered samples were poured into 10-ml acrylic tubes and 20-ml pre-combusted glass ampoules for the Japan Sea and the western North Pacific, respectively, and were then stored frozen in the dark until analysis. The samples of the Japan Sea were collected in duplicate. The detailed results of the bulk fluorescence intensity at a 320-nm excitation and 420-nm emission, the dissolved and total dissolvable Fe concentrations, and the hydrographic parameters for the 5 stations in the Japan Sea have been reported previously^38^.

**Figure 3:**
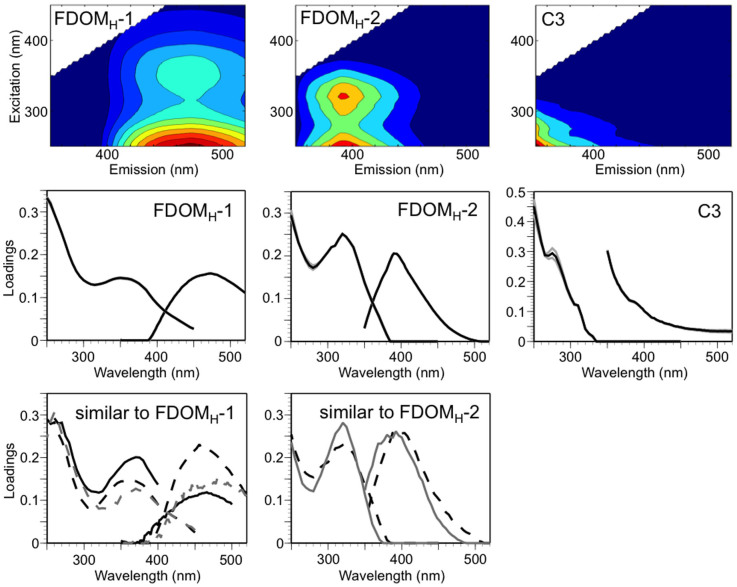
Excitation emission matrix (upper panels) and validation (middle panels) of the three-component model by PARAFAC, and previously published similar PARAFAC components (lower panels). The grey lines in validation plots represent the results from the split half validation analysis. A black solid line, black broken line, grey solid line, and grey broken line in previously published PARAFAC components is from Ref. (23), Ref. (36), Ref. (24), and Ref. (37), respectively.

**Figure 4:**
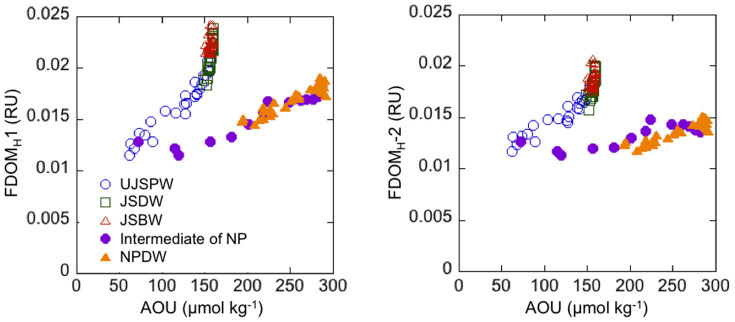
FDOM_H_-1 and FDOM_H_-2 versus AOU in individual water masses of the ocean interior.

Excitation-emission matrix (EEM) fluorescence spectra were obtained using a Horiba Fluoromax-4 fluorometer according to Ref. (48). Samples were thawed and allowed to stand until reaching near room temperature before the EEM measurement. Any residual particles were not observed when samples were thawed to room temperature. Forty-one emission scans from 290 to 550 nm taken at 2-nm intervals were acquired for the excitation wavelengths between 250 and 450 nm at 5-nm intervals. The bandpass was set to 5 nm for both excitation and emission. The fluorescence spectra were scanned with a 0.25-s integration time and acquired in the S/R mode. Several post-acquisition steps were involved in the correction of the fluorescence spectra, including instrumental bias correction and the subtraction of the EEM of Milli-Q water, and the fluorescence units were converted to Raman Units (RU)^49^. The PARAFAC modeling was conducted in MATLAB (Mathworks, Natick, MA) using the DOMFluor toolbox^35^. Contamination from protein-like fluorophores, possibly derived from the acrylic tubes, was evident for samples from the Japan Sea. Thus, the ranges of 250–450 nm and 350–520 nm for excitation and emission, respectively (excluding the wavelength range of the protein-like fluorophore contamination), were used for the PARAFAC modeling. The validation of the PARAFAC model was conducted according to Ref. (35). The spectra of two PARAFAC components, i.e., FDOM_H_-1 and FDOM_H_-2, were compared with those reported in earlier studies through online repository of published PARAFAC components^50^ (Fig. 3). The similarity of components was statistically identified as having Tucker congruence exceeding 0.95. As a result, FDOM_H_-1 was similar to C1 of Ref. (23), C4 of Ref. (36), and C2 of Ref. (37), and FDOM_H_-2 was similar to C4 of Ref. (24) and C6 of Ref. (36). The average of FDOM_H_-1 and FDOM_H_-2 was reported for duplicate sample of the Japan Sea. The difference in fluorescence intensity was 0.0008 ± 0.0007 (n = 112) and 0.0010 ± 0.0008 (n = 112) for FDOM_H_-1 and FDOM_H_-2, respectively.

**Figure 5:**
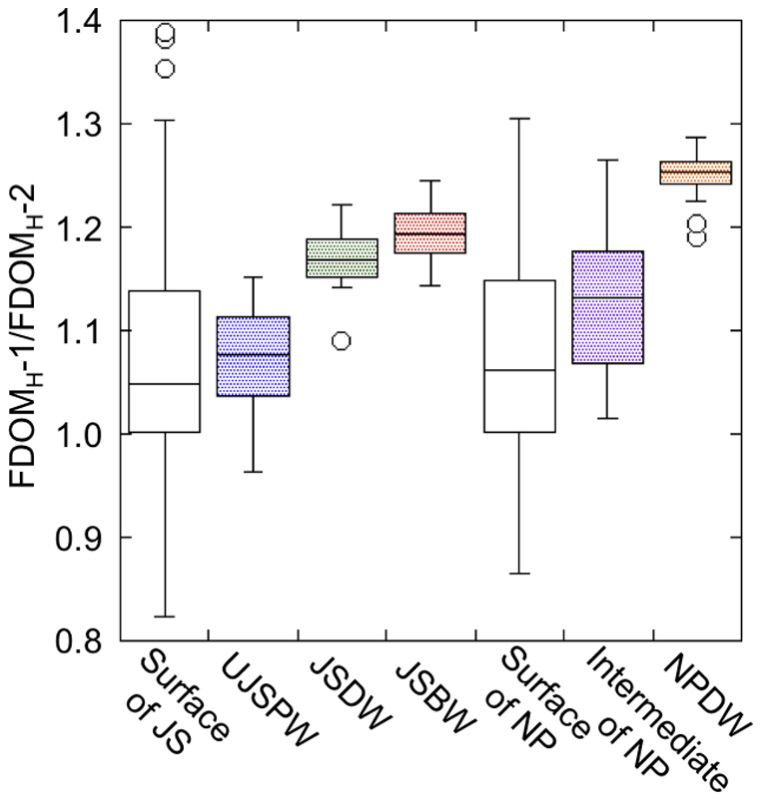
The ratio of FDOM_H_-1 to FDOM_H_-2 for individual water masses. The boxes indicate the interquartile range (25th to 75th percentiles) and include the median value (midline); the whiskers indicate the 10th and 90th percentiles, and the symbols indicate the outliers. Surface of JS and surface of NP means surface waters (<200 m) of the Japan Sea and of the western North Pacific, respectively.

To evaluate any possible effect of contamination, FDOM_H_-1 and FDOM_H_-2, seawater samples were collected from the JSBW (2300–2730 m) at two stations during the T/S *Oshoro-maru* cruise (C253) conducted in April 2013. Seawater samples were collected using Niskin bottles and poured directly into pre-combusted glass vials with teflon-lined caps after triple rinsing to remove any possible contamination from tubes and filters. These samples were stored frozen in the dark until fluorescence analyses. The EEMs of these samples did not show any contamination peaks in the region of the protein-like fluorophores. The fluorescence intensities of FDOM_H_-1 and FDOM_H_-2, and FDOM_H_-1/FDOM_H_-2 ranged from 0.0236–0.0241, 0.0192–0.0197, and 1.22–1.24 (n = 10), respectively. These values are within the same range observed for the JSBW at other stations (0.0215–0.0243, 0.0175–0.0207, and 1.14–1.24 for FDOM_H_-1, FDOM_H_-2, and FDOM_H_-1/FDOM_H_-2, respectively; Figs. 2 and 5), indicating that any contamination that possibly derived from the acrylic tubes did not affect the FDOM_H_ results.

## Author Contributions

Y.Y. contributed to the project planning, with discussion with K.K.; K.K. and K.H. planned the cruises and performed the sampling; K.T. and Y.Y. measured the samples, analyzed the data, and wrote the first draft of the manuscript; all authors contributed to the preparation of the final draft of the manuscript.

## Figures and Tables

**Figure 1:**
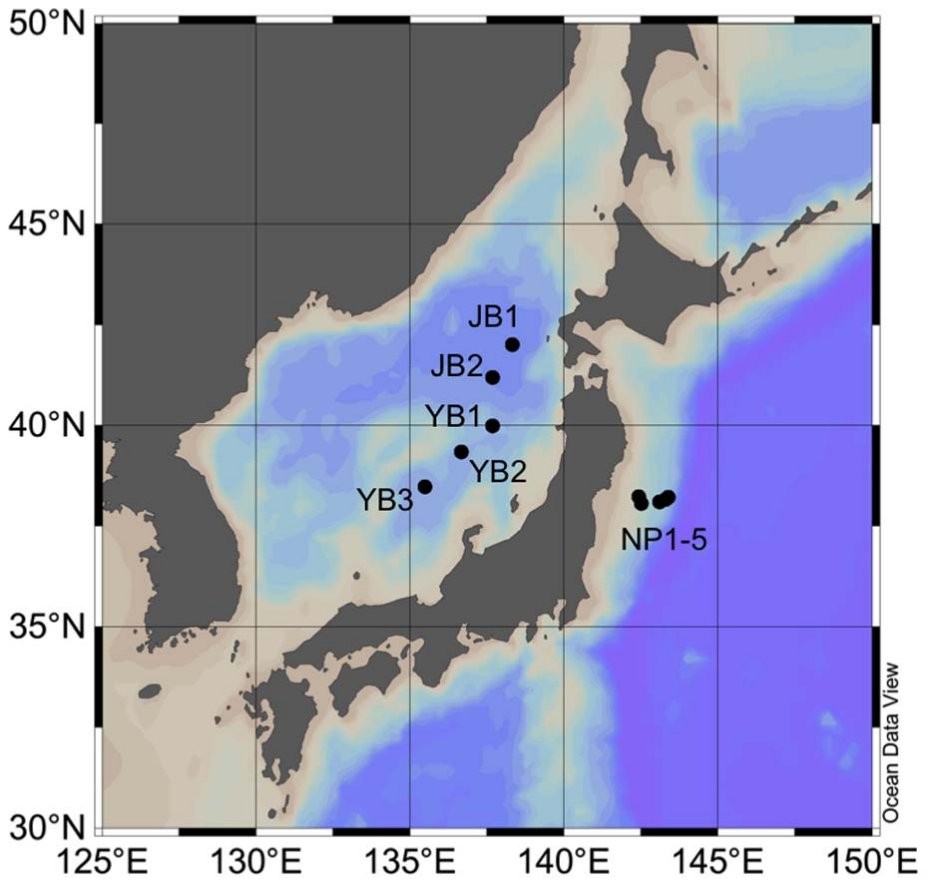
Map of locations sampled for the survey of the FDOM_H_ distribution in the Japan Basin (JB1-2) and the Yamato Basin (YB1-3) of the Japan Sea and the western North Pacific (NP1-5). Map was created using Ocean Data View.

**Table 1:** Relationships between FDOM_H_ and AOU

	FDOM_H_-1 vs AOU				FDOM_H_-2 vs AOU			
	slope	intercept	R^2^	n	slope	intercept	R^2^	n
UJSPW	7.9 × 10^−5^	0.0071	0.92	23	5.3 × 10^−5^	0.0089	0.89	23
JSDW	44 × 10^−5^	−0.047	0.73	25	28 × 10^−5^	−0.026	0.55	25
JSBW		p > 0.05		24		p > 0.05		24
IW of NP	2.9 × 10^−5^	0.0091	0.87	13	1.3 × 10^−5^	0.011	0.62	13
NPDW	3.9 × 10^−5^	0.007	0.88	32	2.7 × 10^−5^	0.0066	0.85	32

The water masses are defined as follows: UJSPW = upper Japan Sea Proper Water (200–1000 m of the Japan Sea), JSDW = Japan Sea Deep Water (1000–2000 m of the Japan Sea), JSBW = Japan Sea Bottom Water (2000 m-bottom of the Japan Sea), IW of NP = intermediate water (200–1000 m) of the western North Pacific, and NPDW = North Pacific Deep Water (1000 m–bottom of the western North Pacific).
